# Pulp Mummification

**Published:** 1907-07

**Authors:** Walter H. Hoyl

**Affiliations:** Dawson, Ga.


### PULP MUMMIFICATION.

By Walter H. Hoyl, Dawson, Ga.

Some teeth are so situated that in my opinion it is impossible for the operator to successfully remove nerves and fill roots to their apices, and I do not doubt there are some conscientious operators today who when meeting a case of this kind resort to the forceps as a proper course of procedure. There are others who spend hours of tedious toil in their endeavors to fill these roots, knowing their efforts are unsuccessful, but trusting that nature by some unknown means will fill the canal he was unable to find. Nature fails him and he too, in the course of a few months, often resorts to the forceps. The writer had this most trying experience, some times feeling as though it was his conscientious duty, when finding an exposed nerve in an inaccessible tooth, advising the patient he did not have the ingenuity to cope with it.

In January, 1905, I had what I call "good luck" showered upon me all at once. I was visiting the office of an elderly practitioner, and while looking over some old dental magazines I noticed in Items of Interest, of December, 1899, a paper on Pulp Mummification by Dr. Owen E. Houghton, of Brooklyn, N. Y. Dr. Houghton's earnestness in advocating this subject impressed me very forcefully, and I am glad to say I took it in as readily as the sponge takes water. I copied the formula and the day following I began to practice pulp mummification, using formula as given by Dr. Houghton with one exception, and I yet have to record my first failure. During this time I have made a record of 163 teeth so treated. During the first few months I confined its use to inaccessible teeth, such as wisdom, distal surface of second molars, occasionally in a six year molar of a child, who would not submit to my thorough removal of nerve and filling roots with gutta percha and cement. With this method it is truly a pleasure to me now to treat that most trying of all cases, the exposure of a nerve in a deciduous tooth. The deciduous teeth are not included, however, in my record of 163 teeth saved. Apparently my success was all that could be desired and I gradually extended its use to all molars, and often to the bicuspids.

I remember in the spring of 1905 while attending dental associations in both Georgia and Florida, I asked some elderly practitioners their opinion of pulp mummification, and invariably they shrugged their shoulders and smiled, saying, if anything at all: "Young man, the less you know of pulp mummification the better off you are." Those same men I now believe made a practice of extracting inaccessible teeth, or were practicing pulp mummification in certain instances. I could never get it into my head that they possessed the ingenuity to fill the roots of all teeth. To them I say this paper is not intended to change their views, I write in hopes that I may reach the ear of some earnest, conscientious, hard-working young dentist who is today spending hours of tedious toil in unsuccessful attempts to fill to the apex roots of posterior teeth. The purport of this paper is accomplished, should I by expressing my views, cause to be saved a single wisdom tooth, or the sixth year molar of some nervous child.

The formula as practiced by Dr. Houghton, and so quoted in his paper, is as follows: Thymol, Dried Alum, Glycerol, equal parts, and Oxide of Zinc, q. s., to make stiff paste.

To this I have added formaline, and my method of proceedure is as follows: Apply arsenic, preferably in the form of a fibre, using a very small quantity and sealing it in the tooth for one week. This form of arsenic is especially advantageous to the dentist practicing in the farming districts where it is seldom convenient for your patient to return to town oftener than once a week. Remove the arsenic, and in 99 per cent. of the cases I find I can remove contents of pulp chamber without pain whatever, using a No. 4 or No. 6 sterilized bur for this purpose, leaving the contents of roots just as I find them. With water syringe, wash out cavity with clean water, dry with alcohol, swab cavity with small piece of cotton saturated with formaline. Take up on your cement spatula enough of paste to fill pulp chamber, incorporate with this a small quantity of formaline. Fill the entire pulp chamber with the paste, sealing it with cement, and completing operation with whatever filling material indicated. Probably the easiest method of applying the paste is to press it into place with a pellet of cotton held in your plyers.

Try this method on the first inaccessible tooth you have, and I believe most firmly that it will be only the matter of a short time when you will have sufficient confidence in it to apply this method to all posterior teeth.

